# Copolymerization of Parylene C and Parylene F to Enhance Adhesion and Thermal Stability without Coating Performance Degradation

**DOI:** 10.3390/polym15051249

**Published:** 2023-02-28

**Authors:** Han Xu, Zhou Yang, Yechang Guo, Qingmei Xu, Songtao Dou, Pan Zhang, Yufeng Jin, Jiajie Kang, Wei Wang

**Affiliations:** 1 Peking University Shenzhen Graduate School, Peking University, Shenzhen 518055, China; 2 School of Integrated Circuits, Peking University, Beijing 100871, China; 3 School of Engineering and Technology, China University of Geosciences (Beijing), Beijing 100083, China; 4 National Key Lab of Micro/Nano Fabrication Technology, Beijing 100871, China; 5 Beijing Advanced Innovation Center for Integrated Circuits, Beijing 100871, China

**Keywords:** Parylene copolymerization, adhesion enhancement, thermal stability, coating performance

## Abstract

Parylene C has been widely used in the fields of microelectromechanical systems (MEMS) and electronic device encapsulation because of its unique properties, such as biocompatibility and conformal coverage. However, its poor adhesion and low thermal stability limit its use in a wider range of applications. This study proposes a novel method for improving the thermal stability and enhancing the adhesion between Parylene and Si by copolymerizing Parylene C with Parylene F. The successful preparation of Parylene copolymer films containing different ratios of Parylene C and Parylene F was confirmed using Fourier-transform infrared spectroscopy and surface energy calculations. The proposed method resulted in the copolymer film having an adhesion 10.4 times stronger than that of the Parylene C homopolymer film. Furthermore, the friction coefficients and cell culture capability of the Parylene copolymer films were tested. The results indicated no degradation compared with the Parylene C homopolymer film. This copolymerization method significantly expands the applications of Parylene materials.

## 1. Introduction

Parylene C has been a widely used polymer in the fields of microelectromechanical systems (MEMS) and electronic device encapsulation because of its unique properties, including pinhole-free conformal coverage and excellent biocompatibility [1,2,3]. However, poor adhesion and low thermal stability have restricted its application [4,5]. As most microfabrication techniques employ Si wafers, the poor adhesion between Parylene C and Si not only affects the process reliability and long-term stability of the prepared device [6,7], but it also necessitates the careful use of chemicals, such as HF and BHF [8]. In addition, Si devices with a Parylene C coating cannot withstand the standard steam sterilization process [9,10].

The current strategies for overcoming the adhesion issues involve both physical and chemical approaches. The physical methods, which aim to increase the contact surface area between Parylene C and Si, include Si surface roughening via reactive ion etching [6] and anchoring Parylene layers on the Si substrate [8]. These procedures unavoidably subject the Si substrate to further processes. The chemical approach consists of adding an adhesion layer, such as the A174 promoter [3], hexamethyldisilazane [11], or molten Parylene C [6,12], or increasing the surface energy by attaching functional groups to the Si substrate [7]. These methods introduce more molecules to the Si substrate and additional complexity to the microfabrication process, possibly affecting device performance.

The thermal stability of Parylene C also limits its application potential. The continuous service temperature of Parylene C is 80 °C in an oxygen environment [13], which is lower than those of most standard MEMS fabrication processes. For example, the standard softbake temperature for photoresists is ~120 °C, whereas soldering and ball bonding typically require temperatures higher than 250 °C [14,15]. It has been reported that Parylene C encapsulation layers delaminate after thermal treatment at 120 °C, rendering it inadequate for processes that include high temperature or thermal accumulation [9].

A number of Parylene variants, such as Parylene N and Parylene AF_4_, have exhibited good thermal stability or better adhesion performances, but have low deposition rates and yields [4,16,17]. Therefore, Parylene with a suitable deposition behavior, adhesion, and thermal stability is urgently required. Low-temperature deposition can improve the deposition rate and yield of Parylene AF_4_; however, this method remains limited by the accompanying stress issues, equipment modifications, and high costs (~30 times those of Parylene C) [17].

In this study, we describe the copolymerization of Parylene C and Parylene F for enhancing the thermal stability and adhesion between Parylene and Si without requiring additional substances except Parylene variants or processes. The coating properties, including the friction coefficient and cell culture capability were investigated and compared with those of the Parylene C homopolymer film to verify its applicability as a coating material.

## 2. Materials and Methods

### 2.1. Copolymerization of Parylene C and Parylene F

**Figure 1:**
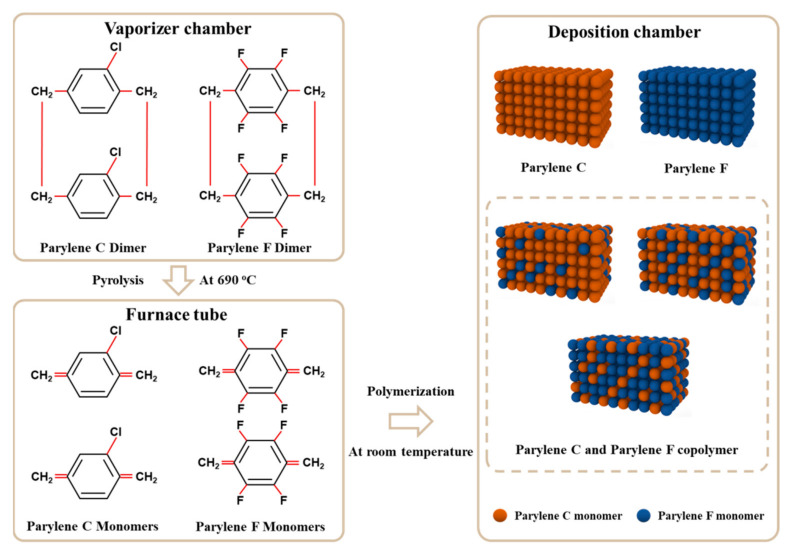
Deposition process of Parylene films. Parylene C and Parylene F dimers are vaporized, pyrolyzed into monomers, and polymerized in the deposition chamber. Parylene homopolymer film (C, F) and Parylene copolymer film (C and F) are illustrated.

Parylene C and F were varied at the substitution groups, as shown in Figure 1. The deposition process was initiated by placing Parylene dimers in the vaporizer of the PDS2010 deposition system. The vaporized dimers then flowed into the furnace, where they were pyrolyzed into monomers. The pyrolysis temperatures for Parylene C and Parylene F were 690 and 720 °C, respectively, to ensure complete decomposition. Subsequently, the Parylene monomers polymerized to produce Parylene films in the room temperature. Parylene C and Parylene F films on Si wafers were fabricated with dimer masses of 1.6 and 4.2 g, respectively, with a deposition pressure of 15 mTorr to guarantee the same thickness deposition chamber at (1 μm).

Parylene C and Parylene F copolymer films were prepared using the same deposition system (SCS PDS2010) and procedures as the Parylene F films. The pyrolysis temperature was set to 720 °C to ensure the complete decomposition of the Parylene F dimers. The deposition pressure was 15 mTorr, and the different mass ratios of the Parylene dimers (C/F) are listed in Table 1. Different dimer mass ratios between Parylene C and Parylene F were conducted to figure out the copolymerization and surface behavior because they are direct and precise. The mass ratio of the deposited film relates to the deposition yield (dimer/thickness), which varies depending on the deposition system and conditions. Dimer mass ratio control was performed based on the thickness of the Parylene film samples maintained at 1 μm.

### 2.2. Traditional Parylene Adhesion Enhancement Methods

Traditional enhancement methods were used to evaluate if the Parylene C-Si adhesion strength met the requirements of the MEMS field. To represent the three approaches to substrate modification, one sample was prepared with the A174 promoter, another with fluorine atoms, and the third with CHF3/SF6 short-time etching. The A174 promoter sample was fabricated using a commercial method that involved immersing the Si wafer in a specific solution (A174:DI water:IPA = 1:100:100, *v*/*v*) for 15 min. The functional groups formed covalent bonds with native SiO_2_ on the Si substrate, working as an interlayer to connect Parylene C and Si [11]. For the second sample, the fluorine atoms of Perfluorodecyltrichlorosilane (FDTS) were deposited on the Si wafer through atomic layer deposition (ALD). The addition of fluorine increased the surface energy of the Si substrate, enhancing the adhesion between the Parylene C and Si. For the third sample, 5 min of reactive ion etching with a CHF_3_ flow rate of 40 sccm and SF_6_ flow rate of 35 sccm was conducted on Si wafers to increase the surface roughness and contact area, enhancing the mechanical adhesion between the Parylene C and Si.

### 2.3. Scratch Test

The Parylene-Si adhesion strength was measured by scratch tests using a nanoindenter (Keysight Model G200, Agilent, Santa Clara, CA, USA) equipped with a Berkovich diamond tip. The scratch test was divided into three stages, including pre-scan, engraved scan, and post-scan. During the pre-scan and post-scan, a constant contact force of 20 μN was applied to the sample surface, and a linear load was applied to the sample surface during the engraved scan, and the scratch speed and loading rate were set to 30 µm/s and 1.8 mN/s, respectively. The scratch length for each sample was 500 µm. A critical load value, friction coefficient with normal load at 1 mN, and lateral force at critical load were recorded. Three samples of each deposited Parylene film were tested during the scratch test, with three tests for each sample.

### 2.4. Fourier-Transform Infrared Spectroscopy

Fourier-transform infrared (FTIR) spectroscopy was used to examine the chemical bonds between the Parylene films and Si substrates. Potential bonds at the Parylene–Si interface and their corresponding wavenumbers include Si-O (1080 cm^−1^) and Si-F (930 cm^−1^) [18]; therefore, the FTIR spectra were measured in the range 1100–900 cm^−1^ (Thermo Scientific Nicolet iS50, Waltham, MA, USA).

### 2.5. Contact Angle Measurements and Surface Energy Calculations

Contact angle measurements were performed with deionized water (DI water) and methanol to characterize the surface energies of the Parylene films. The upper surfaces (exposed to air) and bottom surfaces (in contact with Si) of the deposited Parylene films were assumed to exhibit the same chemical properties, which was verified by the contact angle measurement results of both surfaces. The results were 81.75 ± 0.39° for the upper surfaces and 81.56 ± 1.39° for the bottom surfaces. Therefore, the surface energies of the Parylene films in contact with the Si substrates were obtained by calculating the contact angles of the upper surfaces.

The solid–liquid surface energies were calculated using the contact angle between the liquid and solid substrates, as shown by Young’s equation: [19].

(1) $$\gamma_{sg}=\gamma_{sl}+\gamma_{lg}\cos\varphi$$

where $\gamma_{sg}$, $\gamma_{lg}$, and $\gamma_{sl}$ correspond to the surface energies of the solid substrate in air, liquid in air, and liquid on the solid substrate, respectively. The φ represents the contact angle between the liquid and solid substrates.

Using the Owens–Wendt–Rabel–Kaelble (OWRK) model [20], the surface energy is classified into dispersive and polar components:

(2) $$\gamma_{sl}=\gamma_{sg}+\gamma_{lg}-2\sqrt{\gamma_{sg}^{D}\cdot \gamma_{lg}^{D}}-2\sqrt{\gamma_{sg}^{P}\cdot \gamma_{lg}^{P}}$$

By combining Equations (1) and (2), the surface energy can be rewritten as Equation (3):

(3) $$\gamma_{lg}\left(1+\cos\varphi \right)=2\left(\sqrt{\gamma_{sg}^{D}\cdot \gamma_{lg}^{D}}+\sqrt{\gamma_{sg}^{P}\cdot \gamma_{lg}^{P}}\right)$$

where $\gamma_{sg}^{D}$ and $\gamma_{lg}^{D}$ are the dispersive surface energies of the Parylene film and liquid in air, respectively, and $\gamma_{sg}^{P}$ and $\gamma_{lg}^{P}$ are the polar surface energies of the Parylene film and liquid in air, respectively.

Deionized water and methanol with previously reported dispersive and polar surface energies [20], listed in Table 2, were used to obtain the surface energies of the Parylene films in Equation (3).

Contact angles were measured using a goniometer (SDC-200SH). The Parylene film samples were pre-washed with acetone and ethanol to clean the Parylene surface. Both deionized water and methanol (3 μL) were dispensed on the Parylene film surface of each sample, and three samples of each Parylene film were tested.

### 2.6. Thermal Stability

A synchronous thermal analyzer (Mettler-Toledo TGA/DSC 3+, Greifensee, Switzerland) was used to analyze the thermal stabilities of the Parylene film samples under oxygen and nitrogen atmospheres. The experiment was performed in an alumina crucible at a heating rate of 10 °C/min. The heating ranges of the experiment under oxygen and nitrogen atmospheres were 30–450 °C and 30–600 °C, respectively.

### 2.7. Cell Culture

Mouse lung cancer cells 1601 (separated by collaborator, unpublished) were used to verify the cell culture capabilities of the Parylene films. The 1601 cells were cultured in Dulbecco’s Modified Eagle Medium High Glucose (DMEM High Glucose, Corning) supplemented with 10% fetal bovine serum (FBS), 1 mM sodium pyruvate, 2 mM glutamine, 25 mM HEPES, and 100 U/mL penicillin-streptomycin (Gibco, Thermo Fisher, USA). The 1601 cells were incubated at 37 °C in a humidified atmosphere containing 5% CO_2_. When the confluency reached 80–90%, the cells were trypsinized from the flask and centrifuged at 1000 rpm for 5 min. Next, the 1601 cells (1 × 105 cells/well) were seeded into a Parylene-coated petri dish containing the culture medium (5 mL) for long-term culturing. Petri dishes coated with Parylene C, Parylene F, and Parylene copolymer were tested and cultured for 72 h.

## 3. Results and Discussions

### 3.1. Adhesion Enhancement

**Figure 2:**
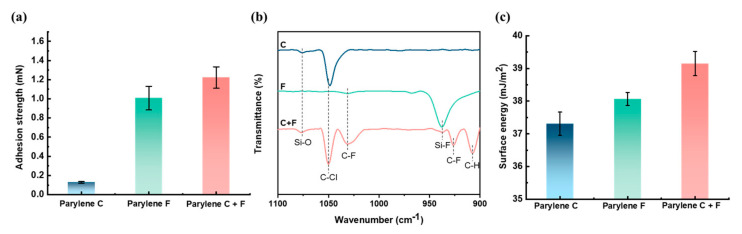
Comparison of the Parylene homopolymer and copolymer films. (**a**) adhesion strength results. (**b**) FTIR results with wavenumber from 1100 to 900 cm^−1^, (**c**) Surface energy results calculated from the contact angles measurements with DI water and Methanol.

The adhesion strengths of Parylene C-Si, Parylene F-Si, and Parylene copolymer (CF)-Si obtained from the FTIR spectra and contact angle measurements are displayed in Figure 2a. The adhesion strengths of the homopolymer Parylene F film and the Parylene CF film increased by factors of 8.3 and 9.55 compared with the Parylene C film, respectively, with the Parylene CF film exhibiting a 22% increase in the adhesion strength compared with the homopolymer Parylene F film. The FTIR spectra in Figure 2b illustrate the bonds between the Parylene films and Si substrates. The Parylene F and Parylene CF films contain Si–F bonds, which contribute to their high adhesion strengths compared with that of the Parylene C film. The Si-O bonds in the Parylene C and Parylene CF samples originate from the native SiO_2_ on the Si substrate and also increase the adhesion strength [21]. The transmittance of the Si–F bonds in the Parylene CF film was lower than that of the Parylene F film, which is consistent with the lower Parylene F dimer mass in the deposition process. However, the adhesion results were affected by the amount of bonds, that is, the Parylene CF film featured fewer Si-F bonds, but exhibited a larger adhesion strength. These results can be partly attributed to an excess of Si-F bonds, which causes mechanical stress concentration [22]. The surface energies of the Parylene films also contribute to the adhesion strength enhancement (Figure 2c). The surface energy of the Parylene CF film is higher than that of the Parylene F film, contributing to the higher adhesion strength. The Parylene C film exhibited the lowest surface energy value among the tested Parylene film samples, along with the unbonded interface, resulting in extremely poor adhesion strength [23].

The adhesion strength enhancement of the Parylene copolymer film was enabled through the combined effects of the generated Si-F bonds and increased surface energy.

**Figure 3:**
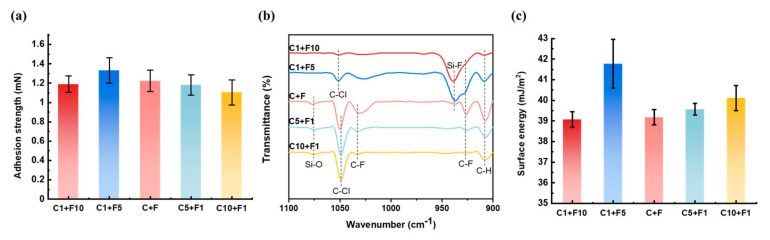
Comparison of the Parylene copolymer films with different dimers mass ratios of Parylene C and Parylene F. (**a**) Adhesion strength. (**b**) FTIR results with wavenumber from 1100 to 900 cm^−1^. (**c**) Surface energy results calculated from the contact angles measurements with DI water and methanol. The generated Si–F bond and change in surface energy explained the principles of the adhesion strength enhancement by the Parylene copolymerization method.

Parylene copolymer films with different mass ratios were analyzed to further investigate the mechanism of the adhesion strength enhancement. The adhesion strength data of Parylene CF films with different Parylene C/Parylene F dimer mass ratios are shown in Figure 3a. Compared with Parylene C, the adhesion strength increases by factors within the range 8.6–10.4 for the Parylene copolymer films, and the increments compared with Parylene F range from 10 to 32%. As the Parylene F/ Parylene C dimer mass ratio decreases from 5 to 0.1 (C1F5, CF, C5F1, and C10F1), the Parylene–Si adhesion strength decreases. This is consistent with the trend of the generated Si-F bonds in Figure 3b, that is, a higher number of bonds enables a larger adhesion strength. The adhesion strength of C1F10 does not conform to this rule, instead presenting a decreased adhesion strength compared with that of the C1F5 film. The FTIR spectra of the Si–O bonds in Figure 3b indicate a saturation of Si-F bonds in the C1F10 and C1F5 films, as no Si-O bonds appear. Further, Figure 3c shows the surface energy of Parylene copolymer films. C1F5 possessed the highest surface energy (41.79 ± 1.18 mJ/m^2^), including the highest polar energy and highest dispersive energy. C1F10 and CF shared similar surface energy (39.08 ± 0.72 and 39.18 ± 0.37 mJ/m^2^). C1F10 has the lowest dispersive energy, which made it the lowest surface energy result of all the Parylene copolymer films. Compared to CF, C5F1 showed slight increase in both polar and dispersive energy, thereby increasing the surface energy (39.56 ± 0.28 mJ/m^2^). C10F1 showed further increased surface energy (40.11 ± 0.61 mJ/m^2^) as the dispersive energy increased compared to C5F1. Therefore, surface energy determines the adhesion strength following Si–F bond saturation. The adhesion strength decreases with the Parylene F mass ratio, whereas the surface energy increases. Therefore, the adhesion strengths of the Parylene films with unsaturated Si–F bonds were dominated by the formation of Si–F bonds rather than the surface energy.

### 3.2. Adhesion Enhancement of Parylene Copolymerization Compared with Traditional Methods

**Figure 4:**
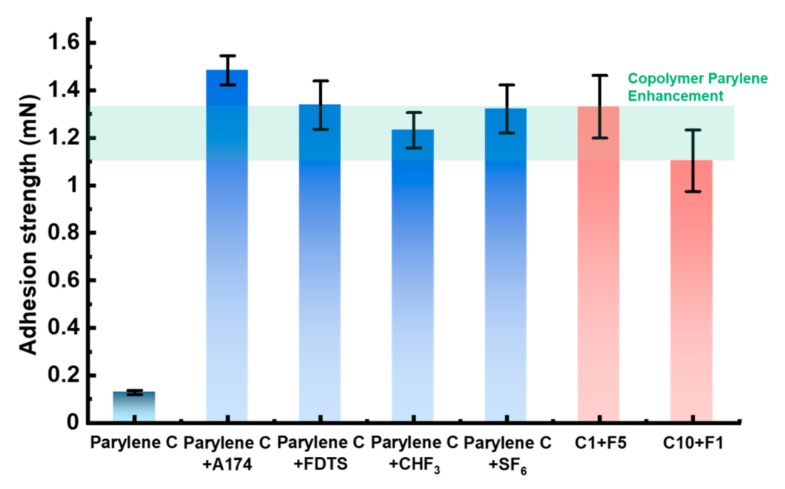
Comparison of the adhesion strength of Parylene-Si by Parylene copolymerization and traditional adhesion enhancement methods. The Parylene copolymer presented comparable enhancement performance to the current methods.

The current methods to enhance the Parylene C–Si adhesion focus on the treatment of the Si substrates. The adhesion strength of the Parylene C-Si substrate obtained from the scratch tests is shown in Figure 4, including those of the A174 promoter, molecular layer epitaxy-deposited FDTS, CHF_3_, and SF_6_ plasma short-time etching samples. Adhesion enhancement with the A174 promoter was considered the standard method, as it has been commercially applied and proven to be sufficiently effective in MEMS applications [3,4,24]. As shown in Figure 4, the adhesion strength is enhanced by a factor of 11.6 with the A174 promoter, which is nearly consistent with the reported results (a factor of 14.8 times, Table 3) [6].

The adhesion strength of the Parylene C-Si substrate with the FDTS pre-treatment is 10.4 times larger than that without treatment. This increase is attributed to the introduction of fluorine atoms, which increase the surface energy of the Si substrate. The CHF_3_ and SF_6_ plasma short-time etching on the Si substrate increase by factors of 9.6 and 10.3, respectively, compared with the untreated Si substrate. The increments are slightly smaller than those of previous XeF_2_ plasma etching results (a factor of 12.1, Table 3) [6].

The results for the Parylene copolymer film are also included in Figure 4. C1F5 and C10F1 exhibit a range of enhanced performances, which are similar to those of the standard method and A174 promoter, and they are comparable with the typical enhancement methods, including FDTS (increasing the polarity of the Si substrate) and CHF_3_/SF_6_ short-time etching on the Si substrate (increasing the contact surface area between the Parylene and Si substrate). With the benefits of the Parylene copolymerization method—simple and without the need for additional substances except Parylene variants and processes—the Parylene–Si adhesion was successfully improved to an acceptable level without affecting the substrate nor imposing additional constraints on subsequent procedures and applications.

### 3.3. Thermal Stability of the Parylene Copolymer Films

**Figure 5:**
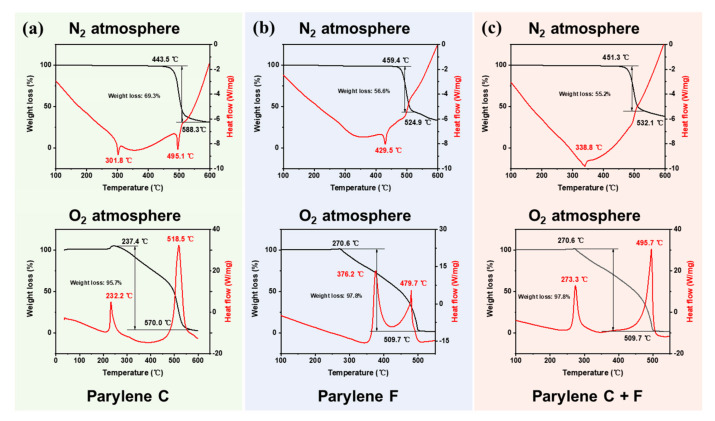
Comparison of the Parylene homopolymer and copolymer films in thermal stability by TGA tests in N_2_ atmosphere and O_2_ atmosphere: (**a**) Parylene C; (**b**) Parylene F; (**c**) Parylene CF. The Parylene copolymer method improved the thermal stability slightly for Parylene films in the N_2_ atmosphere and significantly in the O_2_ atmosphere.

The thermal stabilities of the Parylene films are depicted in Figure 5. The melting temperature, T_m_, is shown in Figure 5a. The T_m_ of the Parylene C film is 301.8 °C, which is identical to the reported values [13,25]. The T_m_ of the Parylene F film is 429.5 °C, and that of the Parylene copolymer film is between Parylene C and Parylene F at 338.8 °C. This increase enables the use of the Parylene film in MEMS standard fabrication techniques that are not accessible to Parylene C, such as ball bonding.

The results of thermal weight loss in an O_2_ atmosphere are shown in Figure 5b. The initial weight loss temperature of Parylene F is 355.6 °C, which is higher than that of Parylene C at 237.4 °C. The Parylene CF film exhibits a higher initial temperature than Parylene C, which is 270.6 °C. Oxidative degradation began with the appearance of the first exothermic peak due to a slight weight increase, and the corresponding temperature values improve from 232.2 °C for the Parylene C film to 273.3 °C for the Parylene CF film.

Therefore, in addition to enhancing the adhesion to Si, the Parylene copolymerization method enables access to a Parylene film with better thermal stability, which is important for MEMS fabrication processes.

### 3.4. Properties of Electronic Device Coating

**Figure 6:**
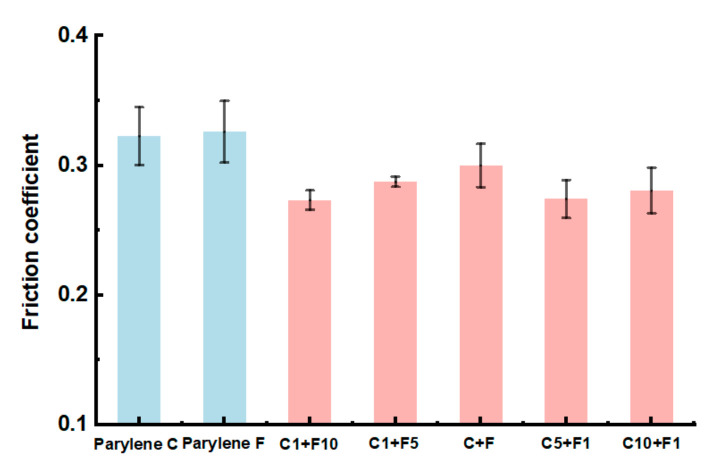
Friction coefficient results of the Parylene homopolymer and copolymer films. The copolymer Parylene films possessed smaller friction coefficient results compared to that of homopolymer Parylene films.

In addition to adhesion and thermal stability, other inherent properties of electronic device coating were analyzed and discussed. Lubricity correlates with the friction coefficient shown in Figure 6. The copolymer Parylene films possessed smaller friction coefficient results compared to that of homopolymer Parylene films. Smaller friction coefficient values indicated better lubricity and smoothness, which may be contributed by the surface heterogeneity during the copolymerization process.

**Figure 7:**
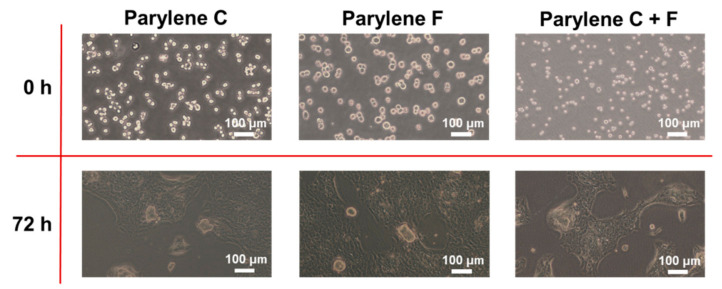
Comparison of the Parylene homopolymer and copolymer films in cell culture. The Parylene C and F copolymer films showed identical biocompatibility to the Parylene C films.

Parylene C is a superior biocompatible material that can function as a cell culture substrate [26,27,28]. The Parylene F and Parylene CF films were compared with the Parylene C films in terms of their cell culture capability, as shown in Figure 7. The Parylene CF film exhibited an identical cell proliferative capability after seeding for 72 h, which indicates that the Parylene copolymer film could replace the Parylene C film as an electronic coating in biological applications.

The cell culture capability of the Parylene CF film, which is comparable with that of Parylene C, along with its smaller friction coefficient, serves the requirements of implantable medical devices and enables an improved biocompatibility to that of Parylene C [28].

## 4. Conclusions

This work proposed a copolymerization method of Parylene C and Parylene F for significantly improving the adhesion to Si and thermal stability, as well as the electronic coating properties, including the friction coefficient and cell culture capability. The mechanism of adhesion enhancement was affected by the number of bonds formed and surface energy. The adhesion was improved by a factor of 10.4, which is comparable with the current enhancement methods. Further, the copolymer exhibited an improved thermal stability (from 301.8 °C to 338.8 °C in a N_2_ atmosphere and from 232.2 °C to 273.3 °C in an O_2_ atmosphere) without degradation, which expands the applications of Parylene materials in the field of MEMS and electronic device coating.

## Figures and Tables

**Table 1:** Dimer mass ratio control of the Parylene C and Parylene F co-deposition.

	C1F10	C1F5	CF	C5F1	C10F1
Dimer mass ratio	Parylene C 0.33 g	Parylene C 0.55 g	Parylene C 1.16 g	Parylene C 1.49 g	Parylene C 1.54 g
	Parylene F 3.3 g	Parylene F 2.75 g	Parylene F 1.16 g	Parylene F 0.30 g	Parylene F 0.15 g

**Table 2:** Surface energy components of DI water and methanol.

	Total Surface Energy (mJ/m^2^)	Dispersive Surface Energy (mJ/m^2^)	Polar Surface Energy (mJ/m^2^)
DI water	72.4	21.1	51.3
Methanol	22.3	17.4	4.9

**Table 3:** Comparison of the adhesion enhancement methods.

Ref	Enhancement Method	Test Method	Enhancement Performance (Multiple Times)	Additional Adhesion Layer	Additional Processes/ Surface Treatment	Wet/ Heating Process
[6]	A174	Peeling test	14.83	Yes	No	Yes
	XeF_2_		12.17	No	Yes	No
	HF clean		11.75	No	Yes	Yes
	Anchoring		13.50	No	Yes	No
	Molten Parylene		12.50	Yes	Yes	Yes
[7]	HF	Peeling test	4.10	No	Yes	Yes
	Hexane		4.04	No	Yes	Yes
	Toluene		4.22	No	Yes	Yes
	P.C.		5.01	No	Yes	Yes
	CF4		1.20	No	Yes	No
[11]	HDMS	Peeling test	~3.20	Yes	No	Yes
This work	A174	Scratch test	11.6	Yes	No	Yes
	FDTS		10.4	Yes	No	Yes
	CHF_3_		9.6	No	Yes	No
	SF_6_		10.3	No	Yes	No
	Parylene Copolymerization		10.40	No	No	No

## Data Availability

The data presented in this study are available on request from the corresponding author.
